# Editorial: Bioinformatics for modern neuroscience

**DOI:** 10.3389/fncom.2024.1385658

**Published:** 2024-02-22

**Authors:** Georgios N. Dimitrakopoulos, Mathieu Di Miceli

**Affiliations:** ^1^Bioinformatics and Human Electrophysiology Laboratory, Department of Informatics, Ionian University, Corfu, Greece; ^2^Worcester Biomedical Research Group, School of Science and the Environment, University of Worcester, Worcester, United Kingdom

**Keywords:** bioinfomatics, neuroscience, editorial, big data, neurology

Bioinformatics relate to applying *in silico* tools to understand biological data. Big data in biology often poses questions on the volume, variety, velocity, veracity, value and variability of data, as well as potential ownership and ethical issues. Researchers will be familiar with (epi)genomics, metabolomics, proteomics, transcriptomics, microbiomics and image-based omics. The current field in neuroscience heavily relies on bioinformatics to decipher the functioning of the nervous system, whether in health or in diseases.

Historically, computation power has been a limiting factor in performing *in silico* biological experiments, which often required supercomputers to run. Thanks to exponential advances in computing, bioinformatics, a division of computational biology, now require computational power that is generally accessible to all researchers, mainly with laptop or desktop computers.

In this Research Topic hosted in *Frontiers in Oncology, Frontiers in Neuroinformatics* and *Frontiers in Computational Neuroscience*, researchers have further widened our understanding of the physiology and pathology of the nervous system, using several approaches. This editorial analyzes and summarizes the articles published in this Research Topic.

With the exponential use of single-cell transcriptomic, several tools are available to analyze transcriptomics data. Qiao designed and validated a new method, titled “factorized linear discriminant analysis” and encoded in Python, which allows correlations to be drawn between gene expression and phenotypic features. The method draws parallels between gene expression and labeled phenotypic datasets, when the latter form Cartesian products of multiple attributes. This method has several applications within and beyond neuroscience, such as the discovery of new cell types, based on clinical features, whether fully or partially annotated.

Bioinformatics can also be used to analyze trends in publications. The glioma research community has been deciphered by Yang et al. in a comprehensive bibliometric analysis. Within more than 3,000 articles published, authors report an exponential annual increase in publication and citation volume on glioma, with China and the USA as leading countries. Interestingly, a clustered network of collaborations has also been observed between researchers, some presenting strong citation bursts, often spanning 2–4 years.

Finding new treatment options for patients not responding to traditional pharmacology is paramount. With single-nucleotide polymorphisms, personalized medicine is key. The current geopolitical climate has war at its core, which can lead to increase of post-traumatic stress disorder (PTSD) cases, a condition which develops after a traumatic event and involves neuroendocrine responses. Gene-drug relationships were scrutinized by Skolariki and Vlamos to optimize personalized treatment for PTSD. After scanning the literature for polymorphisms linked to PTSD, authors identified potential drug candidates using protein-protein and drug-protein interactions. Their results indicate that off-label drugs, which are already in use, appear promising for patients with gene mutations, while clozapine and amrubicin presented interesting pharmacological profiles that could be used to treat PTSD. These methods can also bring new perspectives for other diseases affecting the nervous system, but clinical trials will always be needed to ascertain these initial observations.

Recent epidemiological data suggest that neurodegenerative diseases are becoming more prevalent, with steady increases every year. Protein misfolding appears central in the pathophysiology of such diseases. In proteomics, several tools are currently available to study protein (mis)folding. The advantages and limitations of such tools have been summarized by Krokidis et al.. The authors challenged different computational approaches to predict protein structure, from MODELLER to AlphaFold2 and RoseTTAFold. Several improvements are still needed, for both tools and databases, to improve protein structure predictions and, ultimately, offer new treatment avenues for patients affected with protein-misfolding diseases, especially in neurology.

Medical cannabis has recently gained attention, with the release of cannabis-derived molecules to treat rare forms of epilepsy. Our current understanding of how the endocannabinoid (eCB) system modulates neurotransmission is broadening, but a complete picture has not been fully achieved yet. Using transcriptomics datasets, the results by Cherry et al. further reinforce the role of the eCB system in epilepsy and the response to the psychoactive molecule of cannabis. With the aid of protein-protein databases, the authors also observed interactions of the eCB and dopaminergic systems. Structural analysis of two proteins of the eCB system highlighted conserved residues that are paramount for function.

Thanks to modern computational tools, research in neuroscience is rapidly evolving and will hopefully allow interdisciplinary research to new and exciting questions. Whilst ethical issues are still to be resolved in some specific cases, researchers should embed *in silico* experiments with the more traditional lab-based ones.

## Author contributions

GND: Writing—review & editing. MDM: Writing—original draft, Writing—review & editing.

